# How much are family members satisfied with the intensive care unit in south korea?

**DOI:** 10.1186/2197-425X-3-S1-A657

**Published:** 2015-10-01

**Authors:** J Yoon, G Lim, J Min, G Park, JY Shin, SI Park, YJ Cho, YJ Lee

**Affiliations:** Seoul National University Bundang Hospital, Seongnam-si, Republic of Korea

## Introduction

Meeting the needs of family members of patients in the intensive care unit (ICU) is an important aspect of patient- and family-centered care.

## Objectives

Lack of qualitative research on the ICU experience in South Korea hastens quality improvement. We evaluated the family members ICU experience.

## Methods

We developed a survey asking the family members about their ICU experience, which evaluated the 3 major outcomes: (1)understanding of general information on ICU, (2)understanding of medical status of ICU patient, and (3)satisfaction of information received. The mean score of three outcomes, which represent overall family satisfaction with the ICU care, was calculated for each variable. The study was conducted at the medical ICU from August, 2014 to February, 2015.

## Results

One hundred three surveys were returned. The satisfaction of information received had highest score(79.8 ± 20.6), followed by understanding of medical status of ICU patient(76.0 ± 19.4), and understanding of general information(58.5 ± 23.9). The score of overall family satisfaction was 71.0 ± 16.7. The family members who need financial support and want meetings with social workers and the patient age over 70 year-old were associated with lower overall family satisfaction. The use of renal replacement therapy decreased the understanding of medical status of the ICU patient. The APACHE score over 25 decreased the understanding of general information on ICU. Multi-variate analysis revealed the patients age was significantly associated with decreased overall family satisfaction.

## Conclusions

The age of the patient was an only significant factor associated with decreased family satisfaction. Further researches using validated questionnaire are essential to improve the family members ICU experience.

